# The Risk Factors for Cervical Cytological Abnormalities Among Women Infected With Non-16/18 High-Risk Human Papillomavirus: Cross-sectional Study

**DOI:** 10.2196/38628

**Published:** 2022-12-08

**Authors:** Ting Xiao, Chun-Quan Ou, Jun Yang, Chunhua Wang, Mei Yang, Tingyu Yu, Liang Shen, Xiaohan Xu, Hui Xing

**Affiliations:** 1 State Key Laboratory of Organ Failure Research Department of Biostatistics, School of Public Health Southern Medical University Guangzhou China; 2 School of Public Health Guangzhou Medical University Guangzhou China; 3 Xiangyang Central Hospital Affiliated Hospital of Hubei University of Arts and Science Xiangyang China

**Keywords:** non-16/18 high-risk human papillomavirus, cervical cytological abnormalities, risk factors, logistic regression, cervical cancer, screening, rural, pelvic examination, education, gravidity

## Abstract

**Background:**

High-risk human papillomavirus (hrHPV) infection is a necessary cause of almost all cervical cancers. Relative to hrHPV 16/18 infection, non-16/18 hrHPV infection is of less concern. However, the increasing prevalence of non-16/18 hrHPV infections has become an important public health issue. The early identification and treatment of cervical cytological abnormalities in women infected with non-16/18 hrHPV reduces the incidence of cervical cancer. To date, no study has examined the risk factors for cytological abnormalities in this high-risk population.

**Objective:**

This population-based, cross-sectional study aimed to identify the risk factors for cervical cytological abnormalities in women infected with non-16/18 hrHPV.

**Methods:**

A total of 314,587 women from the general population were recruited for cervical cancer screening at 136 primary care hospitals in Xiangyang, China. Of these, 311,604 women underwent HPV genotyping, and 17,523 non-16/18 hrHPV–positive women were referred for cytological screening according to the screening program. A logistic regression model was used to assess the risk factors for cytological abnormalities among these non-16/18 hrHPV–positive women. A separate analysis was performed to determine the factors influencing high-grade cytological abnormalities.

**Results:**

The non-16/18 hrHPV infection rate was 5.88% (18,323/311,604), which was 3-fold higher than that of hrHPV 16/18 (6068/311,604, 1.95%). Among the non-16/18 hrHPV–positive women who underwent ThinPrep cytologic test, the overall prevalence rates of cervical cytological abnormalities and high-grade cytological abnormalities were 13.46% (2359/17,523) and 1.18% (206/17,523), respectively. Multivariate logistic regression analysis revealed that women with middle or high school educational attainment were at a higher risk of having cytological abnormalities than those who received primary education (odds ratio [OR] 1.31, 95% CI 1.17-1.45; *P*<.001, and OR 1.32, 95% CI 1.14-1.53; *P*<.001, respectively). Living in rural areas (OR 2.58, 95% CI 2.29-2.90; *P*<.001), gravidity ≥3 (OR 2.77, 95% CI 1.19-6.45; *P*=.02), cervix abnormalities detected in pelvic examination (OR 1.22, 95% CI 1.11-1.34; *P*<.001), and having a cervical cancer screening 3 years ago (OR 0.79, 95% CI 0.62-1.00; *P*=.048) were associated with cytological abnormalities. The risk factors for high-grade cytological abnormalities included middle school education (OR 1.45, 95% CI 1.07-1.98; *P*=.02), living in rural regions (OR 1.52, 95% CI 1.10-2.10; *P*=.01), and cervix abnormality (OR 1.72, 95% CI 1.30-2.26; *P*<.001).

**Conclusions:**

The dominant epidemic of non-16/18 hrHPV infection is revealed in Chinese women. Multiple risk factors for cervical cytological abnormalities have been identified in women infected with non-16/18 hrHPV. These findings can provide important information for clinically actionable decisions for the screening, early diagnosis, intervention, and prevention of cervical cancer in non-16/18 hrHPV–positive women.

## Introduction

Globally, cervical cancer is one of the most serious threats to the lives of women. Cervical cancer ranks fourth in terms of both incidence and mortality among women, with an estimated 604,000 new cases and 342,000 deaths globally in 2020 [1]. In China, cervical cancer is a major public health concern because of its high incidence and heavy economic burden [2]. In 2020, it was estimated that there were approximately 110,000 new cases and 59,000 deaths from cervical cancer in China. It is the sixth most frequently diagnosed cancer and the seventh leading cause of cancer-related deaths among Chinese women [3].

Cervical cancer is the most preventable and treatable form of cancer via human papillomavirus (HPV) vaccination, early diagnosis, and effective management. Persistent infection with high-risk HPV (hrHPV) is a necessary but not sufficient cause of almost all cervical cancers [4,5]. There are 14 hrHPV genotypes (16, 18, 31, 33, 35, 39, 45, 51, 52, 56, 58, 59, 66, and 68) that can be detected by real-time polymerase chain reaction assays [6], which are classified as hrHPV 16/18 and non-16/18 hrHPV by current diagnostic paradigms. The majority of cervical cancers are from infection with hrHPV 16, followed by hrHPV 18 [7]. Therefore, hrHPV 16/18 have been recognized as dominant risk factors for cervical cancer and are the focus of medical research, clinical diagnosis, and intervention. As a result, the prevalence of hrHPV 16/18 has significantly decreased over the years [8]. Researchers and the public are relatively less concerned about non-16/18 hrHPV because these infections are considered to be less prevalent and less risky than type 16/18 infections. However, recent studies have reported an increasing prevalence of non-16/18 hrHPV [9,10]. For example, a recent population-based study in China reported a prevalence of 2.2% and 15.3% for hrHPV 16/18 and non-16/18 hrHPV, respectively [11]. The prevalence of non-16/18 hrHPV infection is also a strong predictor of the persistence and progression of cervical diseases [12-15].

Women with cytological abnormalities in the cervix have a relatively high risk of cervical cancer [16]. Early identification and treatment of cervical abnormalities in the early stages or precursor phases of the neoplasm increases the likelihood of lesion regression and reduces the incidence of cervical cancer [17,18]. According to the guidelines of the American Society for Colposcopy and Cervical Pathology [19] and the Chinese Society for Colposcopy and Cervical Pathology [20], women infected with hrHPV 16/18 were directly subjected to colposcopy without cytological screening. Only the women with positive hrHPV genotypes were referred for ThinPrep cytologic test (TCT) followed by colposcopy among those with TCT-positive results. Therefore, following the detection of a non-16/18 hrHPV infection, cytological screening is a useful tool for the selection of women at risk of cervical cancer while reducing the colposcopy burden. A meta-analysis showed that cytological testing in women infected with non-16/18 hrHPV had an overall sensitivity of 69.6% and specificity of 90.2% for detecting cervical intraepithelial neoplasia or worse [21]. However, some women infected with non-16/18 hrHPV may not undergo cytological screening because of inadequate perception of the hazards associated with non-16/18 hrHPV infection or the lack of free screening programs, especially in resource-limited countries. Therefore, identifying the risk factors for cytological abnormalities among those with non-16/18 hrHPV infections will provide important information for impelling those at high risk to undergo screening and ultimately guide clinically actionable decisions for early diagnosis, monitoring, and intervention.

Nevertheless, no previous study has investigated the risk factors for abnormal cytological outcomes in individuals with non-16/18 hrHPV infections [8]. The majority of the previous studies were conducted on the whole population without considering HPV test results, and the factors under study and the conclusions were inconsistent. For example, an observational study in China showed that the risk of cytological abnormalities was associated with HPV genotype [22]. A population-based study in Nigeria showed that demographic characteristics, menopause, gravidity, parity, marital status, and education were associated with cytological abnormalities [23]. Moreover, some previous studies did not find an effect of age on cytological abnormalities in all women or those positive for HPV [24,25]. However, in some studies, the risk of cytological abnormalities significantly increased with age [22,26]. Two studies focused on individuals infected with HPV, among whom education level, years of sexually active life, and parity were risk factors for cytological abnormalities [27,28]. Besides these factors, recent studies have shown an association between cervical cancer and vaginal microbial infection [29,30]. Cervical cancer symptoms, such as bleeding after sex, abnormal vaginal discharge, and pelvic discomfort, may affect the timely diagnosis of cervical cancer [31]. The effects of these factors on cervical cytological abnormalities in individuals with non-16/18 hrHPV infections remain unknown. In particular, the potential impacts of some important factors, including vaginal microbial infection and pelvic examination (PE), on cervical cytological abnormalities have not been investigated previously.

This large population-based study of cervical cancer screening in Chinese women aimed to identify risk factors for cervical cytological abnormalities as well as high-grade cytological abnormalities among women with non-16/18 hrHPV infections, which would provide important information for the screening, early diagnosis, management, and prevention of cervical cancer in the target population (ie, non-16/18 hrHPV–positive women).

## Methods

### Population

The cervical cancer screening program was conducted at 136 primary care hospitals in Xiangyang, China. Participants aged ≥30 years were recruited through media publicity and government notices between January 2017 and February 2018. Women who had received HPV vaccination, were pregnant, had no sexual history, had a hysterectomy, or had a history of pelvic radiotherapy were excluded. All participants were interviewed using questionnaires and underwent PE, vaginal microenvironment test, and HPV genotyping. Women infected with hrHPV 16/18 were directly subjected to colposcopy, whereas women positive for other hrHPV genotypes were referred for TCT, followed by colposcopy in women with TCT-positive results. Histopathological diagnosis was performed if the colposcopy was abnormal or if abnormalities were suspected. A technical manual was developed to regulate the screening process, and the medical staff were trained before the project began.

### Questionnaires

The questionnaire, designed by gynecological oncologists, included age, educational level, residential type (rural or urban), whether the patient is in menopause, age at menopause, family history of cancer, gravidity, parity, contraceptive methods, personal history of other cancers, cervical cancer screening history, and presence of postcoital bleeding and abnormal leucorrhea (Multimedia Appendix 1). Professionally trained clinical staff distributed the questionnaires to the participants and collected data via face-to-face interviews. All data were inputted using the double-entry method.

### PE and Vagina Microenvironment Test

All recruited women underwent routine PE and vaginal microenvironment test. The purpose of the PE was not only to assess pain, bleeding, and vaginal secretions but also to screen for cervical cancer and reproductive tract infections. The PE involved the visual inspection of the vulva, internal speculum examination of the vagina and cervix, and bimanual palpation of the adnexa and uterus. Vaginal secretions were collected with high-vaginal swabs and observed under a microscope to evaluate the vaginal microecosystem, including *Trichomonas vaginalis*, Candida, and Gardnerella [30].

### HPV Genotyping

HPV genotyping was performed using the Cobas HPV test with the Cobas 4800 (Roche Molecular Systems) system, which is approved by the US Food and Drug Administration [6]. Specimens were collected using a cervical brush and sent to the laboratory for professional examination. The Cobas HPV test can provide individual results for hrHPV 16 and hrHPV 18 and simultaneously provide the pooled results for the other 12 non-16/18 hrHPV genotypes (31, 33, 35, 39, 45, 51, 52, 56, 58, 59, 66, and 68).

### TCT Procedure

Women with non-16/18 hrHPV genotypes underwent TCT. The results were reported using the 2001 Bethesda System terminology [32], including negative for intraepithelial lesion or malignancy (NILM); low-grade squamous intraepithelial lesion (LSIL) or high-grade squamous intraepithelial lesion (HSIL); atypical squamous cells of undetermined significance (ASC-US) or atypical squamous cells not possible excluding HSIL (ASC-H); atypical glandular cells (AGC); and squamous cell carcinoma. NILM was considered normal, whereas the others (TCT result worse than ASC-US [ASC-US+]) were considered abnormal.

### Ethics Approval

Ethical approval was obtained from the Ethics Review Committee of Xiangyang Central Hospital, and all procedures followed the ethical standards specified by the institution (approval 2017–004). Written informed consent was obtained from all participants. All examinations complied with the routine medical requirements, and there were provisions for patient safety.

### Statistical Analysis

The enrolled participants were divided into 2 groups based on the TCT results: NILM and ASC-US+. ASC-US+ was considered to be a cervical cytological abnormality. Participants’ characteristics were summarized as counts and percentages, and the chi-square or Fisher exact tests were used to compare whether there were statistical differences in the characteristics between the 2 groups.

Based on the literature and clinical knowledge about the risk factors for cytological abnormalities or cervical cancer, we considered 16 factors that may be associated with cervical cancer. Univariate logistic regression was used to quantify the effect of each factor on the TCT results. Multivariate logistic regression was subsequently performed for all included variables. The generalized variance inflation factor (GVIF) for each variable was calculated to estimate the existence of multicollinearity, and the variable with the largest GVIF^[1 / (2 ×^ *^df^*^)]^ was removed at each step until the GVIF^[1 / (2 ×^ *^df^*^)]^ for all remaining variables was less than 2.24 (ie, 5^1/2^) [33]. Odds ratios (ORs) and their 95% CIs were also calculated. Missing data were not inputted in this study because the rate was low, with 3.41% (597/17,523) of participants having missing values for at least one variable under study.

Since high-grade cytological abnormalities closely associated with cervical cancer require more attention, we specifically identified potential risk factors for high-grade cytological abnormalities (ASC-H, HSIL, AGC, and squamous cell carcinoma) [34] using univariate logistic regression. In the multivariate logistic regression analysis, only variables with *P*<.10 were considered independent variables due to the small sample size. All statistical analyses were performed using R statistical software (version 4.1.1; R Foundation for Statistical Computing). Two-sided statistical tests were used in all analyses, and *P*<.05 was considered statistically significant.

## Results

### Study Subjects

**Figure 1:**
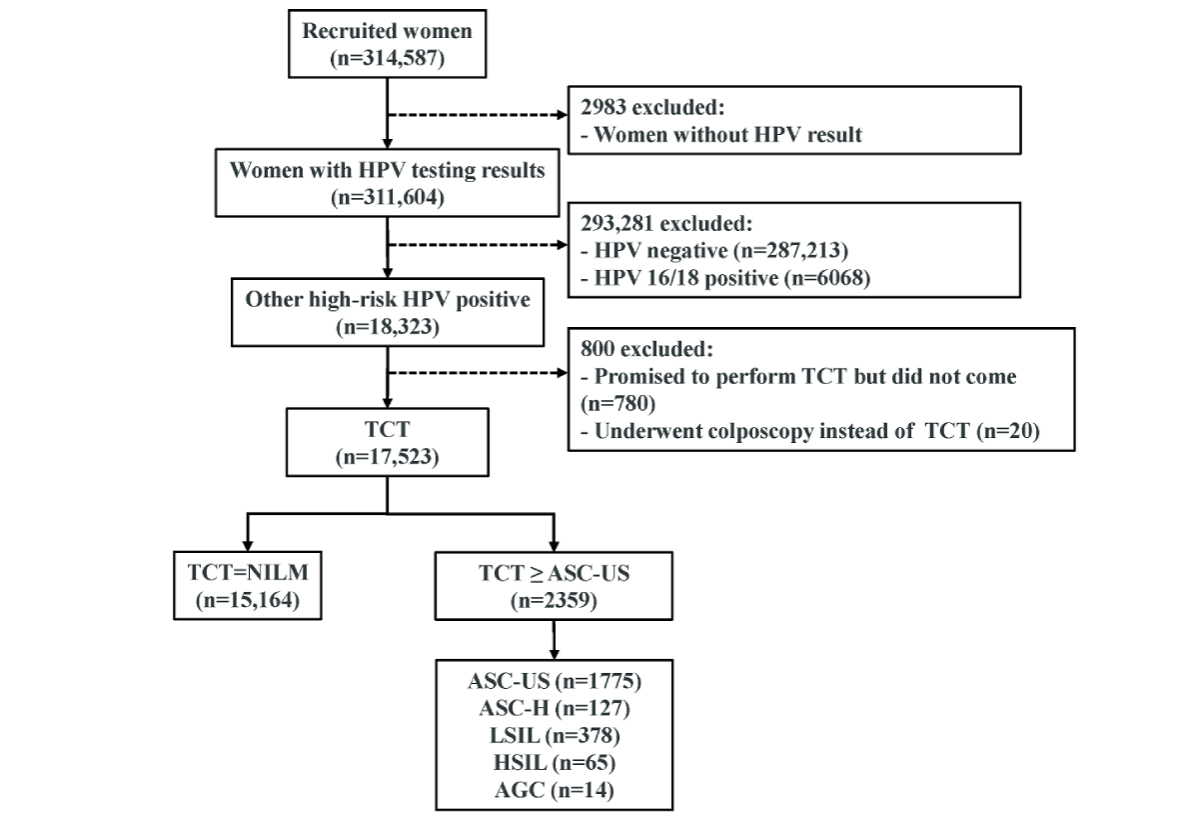
Flow diagram of the identification and selection of study subjects. AGC: atypical glandular cells; ASC-H: atypical squamous cells not possible excluding high-grade squamous intraepithelial lesion; ASC-US: atypical squamous cells of undetermined significance; HPV: human papillomavirus; HSIL: high-grade squamous intraepithelial lesion; LSIL: low-grade squamous intraepithelial lesion; NILM: negative for intraepithelial lesion or malignancy; TCT: ThinPrep cytologic test.

Figure 1 shows the flow of the identification and selection of participants in the study. A total of 311,604 participants in the study underwent HPV genotyping, among which 6068 (1.95%) were infected with hrHPV 16/18, and 18,323 (5.88%) were infected with non-16/18 hrHPV. Of the 18,323 non-16/18 hrHPV–positive participants, 780 (4.26%) promised to receive TCT but did not come back until the end of the program; 20 (0.11%) did not comply with the screening process and underwent colposcopy directly rather than TCT first. As a result, 17,523 participants who were infected with non-16/18 hrHPV and underwent TCT were included in the final analysis of factors associated with cervical abnormalities. The TCT results illustrated that, among them, 15,164 participants (86.54%) had NILM and 2359 (13.46%) had cytologically abnormal findings (ASC-US+). Of the 2359 cytologically abnormal findings, ASC-US was the primary abnormality in TCT (n=1775, 75.25%), followed by LSIL (n=378, 16.02%), ASC-H (n=127, 5.38%), HSIL (n=65, 2.76%), and AGC (n=14, 0.59%).

### Participant Characteristics

Table 1 presents the demographic characteristics and diagnosis-related variables among participants with non-16/18 hrHPV infections. We stratified the participants into 4 groups based on age, and the ages of participants were mainly concentrated in the 40-60 years age range (12,594/17,523, 71.87%). Women with ASC-US+ were relatively less educated than women with NILM (*P*<.001), although 89.78% (15,733/17,523) of participants in both groups had only primary or secondary education. Individuals from rural areas represented the largest proportion of participants with ASC-US+ (1924/2359, 81.56%), with only 62.91% (9540/15,164) of participants with NILM coming from rural areas. A higher proportion of ASC-US+ were participants whose gravidity and parity were ≥3 (1259/2359, 53.37% vs 7219/15,164, 47.6% and 372/2359, 15.77% vs 2147/15,164, 14.16%, respectively). Participants in the ASC-US+ group was less likely to have undergone cervical screening within 3 years or >3 years ago than those in the NILM group (373/2357, 15.83% vs 2674/15,157, 17.64% and 89/2357, 3.78% vs 757/15,157, 4.99%, respectively). Cervix abnormalities detected in PE were more common in participants with ASC-US+ than in those with NILM (1020/2346, 43.48% vs 5678/15,067, 37.69%, respectively). There were no statistically significant differences in other factors between the 2 groups.

**Table 1:** Demographic characteristics and diagnosis-related variables for participants with non-16/18 high-risk human papillomavirus infection.

Characteristics		Overall, n (%)	Groups		*P* value	
			NILM^a^, n (%)	ASC-US+^b^, n (%)		
**Age (years; overall: n=17,523; NILM: n=15,164; ASC-US+: n=2359)**						.84
	<40	2670 (15.24)	2302 (15.18)	368 (15.6)		
	40-50	5817 (33.2)	5028 (33.16)	789 (33.45)		
	50-60	6777 (38.67)	5884 (38.8)	893 (37.85)		
	≥60	2259 (12.89)	1950 (12.86)	309 (13.1)		
**BMI^c,d^ (overall: n=17,359; NILM: n=15,023; ASC-US+: n=2336)**						.44
	Normal	12,551 (72.3)	10,858 (72.28)	1693 (72.47)		
	Underweight	699 (4.03)	595 (3.96)	104 (4.45)		
	Overweight	4109 (23.67)	3570 (23.76)	539 (23.07)		
**Education (overall: n=17,523; NILM: n=15,164; ASC-US+: n=2359)**						<.001
	Primary	8439 (48.16)	7350 (48.47)	1089 (46.16)		
	Middle	4896 (27.94)	4126 (27.21)	770 (32.64)		
	High	2398 (13.68)	2071 (13.66)	327 (13.86)		
	Graduate	1790 (10.22)	1617 (10.66)	173 (7.34)		
**Region (overall: n=17,523; NILM: n=15,164; ASC-US+: n=2359)**						<.001
	Urban	6059 (34.58)	5624 (37.09)	435 (18.44)		
	Rural	11,464 (65.42)	9540 (62.91)	1924 (81.56)		
**Family history of cancer (overall: n=17,523; NILM: n=15,164; ASC-US+: n=2359)**						.27
	No	17,170 (97.99)	14,866 (98.03)	2304 (97.67)		
	Yes	353 (2.01)	298 (1.97)	55 (2.33)		
**Menopause (overall: n=17,523; NILM: n=15,164; ASC-US+: n=2359)**						.43
	No	9031 (51.54)	7797 (51.42)	1234 (52.31)		
	Yes	8492 (48.46)	7367 (48.58)	1125 (47.69)		
**Gravidity (overall: n=17,523; NILM: n=15,164; ASC-US+: n=2359)**						<.001
	0	547 (3.12)	497 (3.28)	50 (2.12)		
	1-2	8498 (48.5)	7448 (49.12)	1050 (44.51)		
	≥3	8478 (48.38)	7219 (47.6)	1259 (53.37)		
**Parity (overall: n=17,523; NILM: n=15,164; ASC-US+: n=2359)**						.005
	0	602 (3.43)	543 (3.58)	59 (2.5)		
	1-2	14,402 (82.19)	12,474 (82.26)	1928 (81.73)		
	≥3	2519 (14.38)	2147 (14.16)	372 (15.77)		
**Cervical screening^c^ (overall: n=17,514; NILM: n=15,157; ASC-US+: n=2357)**						.002
	Never	13,621 (77.77)	11,726 (77.36)	1895 (80.4)		
	Within 3 years	3047 (17.4)	2674 (17.64)	373 (15.83)		
	>3 years ago	846 (4.83)	757 (4.99)	89 (3.78)		
**History of other cancers (overall: n=17,523; NILM: n=15,164; ASC-US+: n=2359)**						.45
	No	16,954 (96.75)	14,665 (96.71)	2289 (97.03)		
	Yes	569 (3.25)	499 (3.29)	70 (2.97)		
**Postcoital bleeding (overall: n=17,523; NILM: n=15,164; ASC-US+: n=2359)**						.32
	No	17,369 (99.12)	15,026 (99.09)	2343 (99.32)		
	Yes	154 (0.88)	138 (0.91)	16 (0.68)		
**Abnormal leukorrhea (overall: n=17,523; NILM: n=15,164; ASC-US+: n=2359)**						.92
	No	16,471 (94)	14,252 (93.99)	2219 (94.07)		
	Yes	1052 (6)	912 (6.01)	140 (5.93)		
**PE^e^: cervix abnormality^c^ (overall: n=17,413; NILM: n=15,067; ASC-US+: n=2346)**						<.001
	Normal	10,715 (61.53)	9389 (62.31)	1326 (56.52)		
	Abnormal	6698 (38.47)	5678 (37.69)	1020 (43.48)		
**Trichomonas infection^c^ (overall: n=16,926; NILM: n=14,629; ASC-US+: n=2297)**						.96
	No	16,411 (96.96)	14,183 (96.95)	2228 (97)		
	Yes	515 (3.04)	446 (3.05)	69 (3)		
**Candida infection^c^ (overall: n=16,926; NILM: n=14,629; ASC-US+: n=2297)**						.97
	No	16,159 (95.47)	13,967 (95.47)	2192 (95.43)		
	Yes	767 (4.53)	662 (4.53)	105 (4.57)		
**Gardnerella infection^c^ (overall: n=16,926; NILM: n=14,629; ASC-US+: n=2297)**						.54
	No	16,858 (99.6)	14,568 (99.58)	2290 (99.7)		
	Yes	68 (0.4)	61 (0.42)	7 (0.3)		

^a^NILM: negative for intraepithelial lesion or malignancy.

^b^ASC-US+: ThinPrep cytologic test result worse than atypical squamous cells of undetermined significance.

^c^The sum does not equal the total number because of the existence of missing values.

^d^BMI categories: underweight (<18.5), normal (18.5-25), and overweight (≥25).

^e^PE: pelvic examination.

### The Risk Factors for Cytological Abnormalities

**Figure 2:**
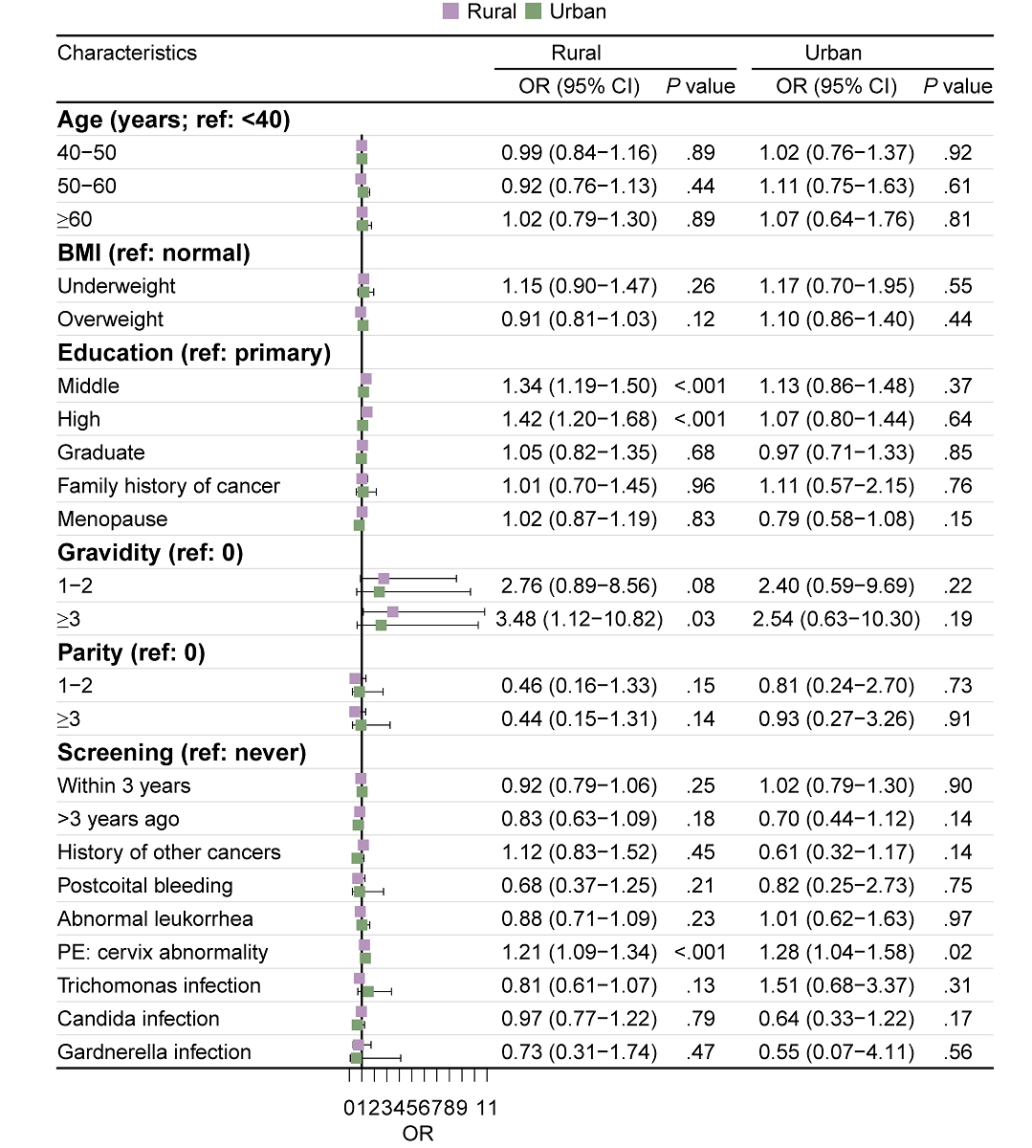
Multivariate logistic regression analysis stratified on area to explore risk factors for cytological abnormalities among individuals infected with non-16/18 high-risk human papillomavirus. OR: odds ratio; PE: pelvis examination.

Table 2 shows the results of the univariate and multivariate logistic regression, which assessed the risk factors of ASC-US+ for participants with non-16/18 hrHPV. A higher incidence of ASC-US+ was observed in women who attended middle or high school (OR 1.31, 95% CI 1.17-1.45; *P*<.001, and OR 1.32, 95% CI 1.14-1.53; *P*<.001, respectively) and those living in rural areas (OR 2.58, 95% CI 2.29-2.90; *P*<.001). The likelihood of ASC-US+ increased with gravidity ≥3 (OR 2.77, 95% CI 1.19-6.45; *P*=.02) and cervix abnormalities detected in PE (OR 1.22, 95% CI 1.11-1.34; *P*<.001). The risk of ASC-US+ was lower in the women who had cervical screening >3 years ago (OR 0.79, 95% CI 0.62-1.00; *P*=.048) than in those with no previous screening. When stratified by rural or urban areas, the results showed that middle or high school education (OR 1.34, 95% CI 1.19-1.50; *P*<.001, and OR 1.42, 95% CI 1.20-1.68; *P*<.001, respectively) and gravidity ≥3 (OR 3.48, 95% CI 1.12-10.82; *P*=.03) were associated with significantly increased risk in women living in rural areas. Cervix abnormalities detected in PE was associated with an increased risk for ASC-US+ in both rural (OR 1.21, 95% CI 1.09-1.34; *P*<.001) and urban (OR 1.28, 95% CI 1.04-1.58; *P*=.02) areas (Figure 2).

**Table 2:** Risk factors of ASC-US+^a^ for participants with non-16/18 high-risk human papillomavirus infection explored by univariate and multivariate logistic regression.

Characteristics		Univariate logistic		Multivariate logistic			
		OR^b^ (95% CI)	*P* value	Full model^c^		Simplified model^d^	
				OR (95% CI)	*P* value	OR (95% CI)	*P* value
**Age (years; ref^e^: <40)**							
	40-50	0.98 (0.86-1.12)	.79	0.99 (0.86-1.14)	.87	N/A^f^	N/A
	50-60	0.95 (0.83-1.08)	.44	0.96 (0.80-1.14)	.61	N/A	N/A
	≥60	0.99 (0.84-1.17)	.92	1.02 (0.82-1.28)	.83	N/A	N/A
**BMI^g^ (ref: normal)**							
	Underweight	1.12 (0.90-1.39)	.30	1.15 (0.93-1.44)	.21	N/A	N/A
	Overweight	0.97 (0.87-1.07)	.54	0.94 (0.85-1.05)	.28	N/A	N/A
**Education (ref: primary)**							
	Middle	1.26 (1.14-1.39)	<.001	1.31 (1.17-1.45)	<.001	1.30 (1.18-1.44)	<.001
	High	1.07 (0.93-1.22)	.35	1.32 (1.14-1.53)	<.001	1.35 (1.18-1.56)	<.001
	Graduate	0.72 (0.61-0.86)	<.001	1.05 (0.87-1.27)	.61	1.09 (0.91-1.31)	.35
**Region (ref: urban)**							
	Rural	2.61 (2.34-2.91)	<.001	2.58 (2.29-2.90)	<.001	2.60 (2.32-2.91)	<.001
Family history of cancer		1.19 (0.89-1.59)	.24	1.04 (0.76-1.43)	.80	N/A	N/A
Menopause		0.96 (0.88-1.05)	.42	0.97 (0.84-1.11)	.65	N/A	N/A
**Gravidity (ref: 0)**							
	1-2	1.40 (1.04-1.89)	.03	2.28 (0.98-5.29)	.06	2.17 (0.99-4.78)	.05
	≥3	1.73 (1.29-2.33)	<.001	2.77 (1.19-6.45)	.02	2.67 (1.21-5.88)	.02
**Parity (ref: 0)**							
	1-2	1.42 (1.08-1.87)	.01	0.61 (0.28-1.32)	.21	0.56 (0.27-1.16)	.12
	≥3	1.59 (1.19-2.13)	.002	0.61 (0.28-1.35)	.23	0.56 (0.27-1.18)	.13
**Screening (ref: never)**							
	Within 3 years	0.86 (0.77-0.97)	.02	0.94 (0.83-1.07)	.36	0.94 (0.83-1.07)	.34
	>3 years ago	0.73 (0.58-0.91)	.006	0.79 (0.62-1.00)	.048	0.81 (0.64-1.01)	.07
History of other cancers		0.90 (0.70-1.16)	.410	0.97 (0.74-1.28)	.85	N/A	N/A
Postcoital bleeding		0.74 (0.44-1.25)	.264	0.71 (0.41-1.23)	.22	N/A	N/A
Abnormal leukorrhea		0.99 (0.82-1.18)	.88	0.89 (0.74-1.08)	.26	N/A	N/A
PE^h^: cervix abnormality		1.27 (1.16-1.39)	<.001	1.22 (1.11-1.34)	<.001	1.23 (1.13-1.35)	<.001
Trichomonas infection		0.98 (0.76-1.27)	.91	0.85 (0.65-1.10)	.22	N/A	N/A
Candida infection		1.01 (0.82-1.25)	.92	0.91 (0.73-1.13)	.40	N/A	N/A
Gardnerella infection		0.73 (0.33-1.60)	.43	0.69 (0.31-1.52)	.36	N/A	N/A

^a^ASC-US+: ThinPrep cytologic test result worse than atypical squamous cells of undetermined significance.

^b^OR: odds ratio.

^c^Full model: including all variables.

^d^Simplified model: including the variables with *P*<.10 in the univariate logistic regression.

^e^ref: reference.

^f^N/A: not applicable.

^g^BMI categories: underweight (<18.5), normal (18.5-25), and overweight (≥25).

^h^PE: pelvic examination.

### The Risk Factors for High-Grade Cytological Abnormalities

Table 3 shows the risk factors for high-grade cytological abnormalities. Education, region, cervical screening, and cervix abnormalities detected in PE were included in the multivariate analysis as their *P* values were <.10 in the univariate analysis. Among these factors, significant differences were observed with middle school education (OR 1.45, 95% CI 1.07-1.98; *P*=.02), rural region (OR 1.52, 95% CI 1.10-2.10; *P*=.01), and cervix abnormality (OR 1.72, 95% CI 1.30-2.26; *P*<.001).

**Table 3:** Risk factors of high-grade cytological abnormalities for participants with non-16/18 high-risk human papillomavirus infection explored by univariate and multivariate logistic regression.

Characteristics		Univariate logistic			Multivariate logistic				
		OR^a^ (95% CI)	*P* value	Full model^b^				Simplified model^c^	
				OR (95% CI)		*P* value	OR (95% CI)		*P* value
**Age (years; ref^d^: <40)**									
	40-50	0.98 (0.86-1.12)	.79	0.99 (0.86-1.14)		.87	N/A^e^		N/A
	50-60	0.95 (0.83-1.08)	.44	0.96 (0.80-1.14)		.61	N/A		N/A
	≥60	0.99 (0.84-1.17)	.92	1.02 (0.82-1.28)		.83	N/A		N/A
**BMI^f^ (ref: normal)**									
	Underweight	1.12 (0.90-1.39)	.30	1.15 (0.93-1.44)		.21	N/A		N/A
	Overweight	0.97 (0.87-1.07)	.54	0.94 (0.85-1.05)		.28	N/A		N/A
**Education (ref: primary)**									
	Middle	1.26 (1.14-1.39)	<.001	1.31 (1.17-1.45)		<.001	1.30 (1.18-1.44)		<.001
	High	1.07 (0.93-1.22)	.35	1.32 (1.14-1.53)		<.001	1.35 (1.18-1.56)		<.001
	Graduate	0.72 (0.61-0.86)	<.001	1.05 (0.87-1.27)		.61	1.09 (0.91-1.31)		.35
**Region (ref: urban)**									
	Rural	2.61 (2.34-2.91)	<.001	2.58 (2.29-2.90)		<.001	2.60 (2.32-2.91)		<.001
Family history of cancer		1.19 (0.89-1.59)	.24	1.04 (0.76-1.43)		.80	N/A		N/A
Menopause		0.96 (0.88-1.05)	.42	0.97 (0.84-1.11)		.65	N/A		N/A
**Gravidity (ref: 0)**									
	1-2	1.40 (1.04-1.89)	.03	2.28 (0.98-5.29)		.06	2.17 (0.99-4.78)		.05
	≥3	1.73 (1.29-2.33)	<.001	2.77 (1.19-6.45)		.02	2.67 (1.21-5.88)		.02
**Parity (ref: 0)**									
	1-2	1.42 (1.08-1.87)	.01	0.61 (0.28-1.32)		.21	0.56 (0.27-1.16)		.12
	≥3	1.59 (1.19-2.13)	.002	0.61 (0.28-1.35)		.23	0.56 (0.27-1.18)		.13
**Screening (ref: never)**									
	Within 3 years	0.86 (0.77-0.97)	.02	0.94 (0.83-1.07)		.36	0.94 (0.83-1.07)		.34
	>3 years ago	0.73 (0.58-0.91)	.006	0.79 (0.62-1.00)		.048	0.81 (0.64-1.01)		.07
History of other cancers		0.90 (0.70-1.16)	.410	0.97 (0.74-1.28)		.85	N/A		N/A
Postcoital bleeding		0.74 (0.44-1.25)	.264	0.71 (0.41-1.23)		.22	N/A		N/A
Abnormal leukorrhea		0.99 (0.82-1.18)	.88	0.89 (0.74-1.08)		.26	N/A		N/A
PE^g^: cervix abnormality		1.27 (1.16-1.39)	<.001	1.22 (1.11-1.34)		<.001	1.23 (1.13-1.35)		<.001
Trichomonas infection		0.98 (0.76-1.27)	.91	0.85 (0.65-1.10)		.22	N/A		N/A
Candida infection		1.01 (0.82-1.25)	.92	0.91 (0.73-1.13)		.40	N/A		N/A
Gardnerella infection		0.73 (0.33-1.60)	.43	0.69 (0.31-1.52)		.36	N/A		N/A

^a^OR: odds ratio.

^b^Full model: including all variables.

^c^Simplified model: including the variables with *P*<.10 in the univariate logistic regression.

^d^ref: reference.

^e^N/A: not applicable.

^f^BMI categories: underweight (<18.5), normal (18.5-25), and overweight (≥ 25).

^g^PE: pelvic examination.

## Discussion

### Principal Findings

Middle or high school education, living in rural areas, gravidity ≥3, and cervix abnormalities detected in PE were the risk factors for ASC-US+ in this study. In addition, receiving cervical screening >3 years ago was negatively associated with the prevalence of ASC-US+ among women with non-16/18 hrHPV infections. Our findings may have important implications for the prevention and control of cervical cancer in non-16/18 hrHPV–positive individuals. High-risk groups identified by their risk factors should be carefully diagnosed and treated according to medical advice to prevent adverse outcomes.

We observed that age had no effect in this study. Considering the large sample size of this study (n=17,523) and broad age range (from 30 to 78 years old), we believe that the result of null effect of age on cytological abnormalities in women infected with non-16/18 hrHPV is reliable. Some previous studies also did not find an effect of age on cytological abnormalities in all women or those infected with HPV [24,25,28]. However, in some studies, the risk of cytological abnormalities increased significantly with age [22,26]. This inconsistency may be due to differences in race, social environment, behavior, and habits in different areas.

Education was an important risk factor for cytological abnormalities. Women with middle and high school education were more likely to have cytological abnormalities than those with primary school education. Previous studies have also shown that women with middle and high school education are at a higher risk for cervical cancer [4,27,35]. The reason may be that women with primary school education tend to marry earlier and have more stable sexual partners. Previous studies have reported that both women and their husbands’ lifetime number of sexual partners were significantly positively correlated with cervical cancer risk [36].

Women in rural areas had a higher probability of cytological abnormalities. Poor sanitation, insufficient knowledge about cervical cancer, and poor awareness of prevention in rural areas [37] could increase vulnerability to cervical cancer. In addition, women in rural areas have a lower frequency of gynecologic examination and cervical cancer screening than those in urban areas [38], resulting in an inability to detect abnormalities and receive timely treatment. Therefore, efforts should be intensified in rural areas to popularize cervical cancer prevention knowledge and reduce the incidence of cervical cancer. Furthermore, risk factors for cytological abnormalities differ in rural and urban areas. Among rural women, middle or high school education and gravidity ≥3 were associated with an increased risk of cytological abnormalities, whereas such results were not observed in urban women. This finding means that narrowing and eventually addressing the socioeconomic gap is imperative for cervical cancer prevention.

The prevalence of cytological abnormalities significantly increased when gravidity was ≥3, which may be related to hormonal changes during pregnancy [39]. Female sex hormones (estrogen and progesterone) may affect immune function [40]. Unstable sex hormone levels reduce immunity in women, thus lowering the resistance to hrHPV, weakening the ability to clear hrHPV, and resulting in an increased probability of cytological abnormalities. Women with high gravidity who are infected with hrHPV are recommended to consult their physician for further diagnosis in a timely manner. In addition to complying with the cervical cancer screening guidelines [41], it is recommended that women who are infected with non-16/18 hrHPV undergo HPV examination and cytology test again 1 year later, even if their TCT results were NILM.

Women with cervix abnormalities in PE are more likely to have cytological abnormalities. Previous studies have shown that the appearance of the cervix is correlated with the incidence of cervical cancer [42]. In the United Kingdom, both clinical practice guidelines on the diagnosis of cancer [43] and the National Institute for Health and Care Excellence guidelines [44] recommend visualizing the cervix to facilitate timely diagnosis of women with cervical cancer. Although no such guidelines exist in the United States, the American College of Obstetricians and Gynecologists Committee on Gynecologic Practice suggests a similar approach [45]. Therefore, PE is recommended to be added to the physical examination in women to detect the abnormal appearance of the cervix and facilitate early treatment, thereby lowering the incidence of cervical cancer.

Some cohort studies have shown that cervical cytology screening can reduce the incidence of cervical cancer by detecting precancerous lesions and early-stage cancer [18,46]. We found that cervical screening performed >3 years ago was a protective factor against cytological abnormalities. However, such protective effects were not observed when screening was performed within 3 years. Women with cytological abnormalities are particularly recommended to undergo regular follow-up cytological screening to monitor the progression or regression of cervical abnormalities. Women who screened for cervical cancer within 3 years were more likely to have previous cervical abnormalities than those screened >3 years ago. Further, women who were screened for cervical cancer >3 years ago were likely to have normal results on their last cervical cancer examination, indicating a low risk of current cytological abnormalities. Undoubtedly, well-organized screening programs have been documented to reduce the incidence and mortality of cervical cancer [17,47,48]. Women are advised to adhere to the Cervical Cancer Screening Program, which is expected to expand worldwide. It is recommended that women with non-16/18 hrHPV–positive status undergo regular cervical cancer screenings regardless of disease status and follow up with doctors if abnormalities are detected upon screening.

### Comparison With Prior Work

To the best of our knowledge, this is the first study investigating cytological abnormalities in women infected with non-16/18 hrHPV. A few previous studies have explored the influencing factors of cytological abnormalities in all women; however, they did not focus on this overlooked subpopulation of those infected with non-16/18 hrHPV. Compared with previous studies, one of the strengths of this study is the large sample size of 17,523 individuals collected from multiple centers, which guarantees high statistical power and good precision of the estimates. In addition, we considered other potential influencing factors, including demographic characteristics, menstruation and fertility, PE results, and vaginal microenvironment infection.

### Limitations

Our study has some limitations. First, this was a cross-sectional study without detailed information from previous screening results, and all subjects were infected with non-16/18 hrHPV detected by the current screening. The HPV genotype was not considered in this study because the selected Cobas HPV test could not detect specific types of non-16/18 hrHPV. This information on specific HPV genotypes and the persistence of infection may have an impact on abnormalities according to previous research [27,49]. Second, this study included only Chinese women; the risk factors for cytological abnormalities may differ according to ethnicity, social environment, and behavioral habits. Therefore, caution should be exercised when extrapolating the conclusions to other populations. Third, personal behaviors, such as cigarette smoking and long-term oral contraceptive use, which have been proven to be cofactors in cervical cancer [50], were not controlled in our study. As a result, the relationship between these factors and cytological abnormalities could not be investigated. Finally, reporting and recall biases may exist because of the use of a self-reported questionnaire.

### Conclusion

This large-scale, cross-sectional study assessed the prevalence and risk factors of cytological abnormalities in 17,523 Chinese women infected with non-16/18 hrHPV. Middle or high school education, living in rural areas, gravidity ≥3, and cervix abnormalities detected in PE were found to be risk factors for cytological abnormalities, whereas receiving cervical screening >3 years ago was associated with a reduced prevalence of cytological abnormalities. In addition, middle school education, living in rural regions, and cervix abnormality were risk factors for high-grade cytological abnormalities. More attention should be paid to improving diagnostic, management, and vaccination strategies among individuals with non-16/18 hrHPV infections.

## Appendix

Multimedia Appendix 1 The questionnaire in both English and Chinese.

## Abbreviations

AGC: atypical glandular cells
ASC-H: atypical squamous cells not possible excluding high-grade squamous intraepithelial lesion
ASC-US: atypical squamous cells of undetermined significance
ASC-US+: ThinPrep cytologic test result worse than atypical squamous cells of undetermined significance
GVIF: generalized variance inflation factor
HPV: human papillomavirus
hrHPV: high-risk human papillomavirus
HSIL: high-grade squamous intraepithelial lesion
LSIL: low-grade squamous intraepithelial lesion
NILM: negative for intraepithelial lesion or malignancy
OR: odds ratio
PE: pelvic examination
TCT: ThinPrep cytologic test

## Data Availability

The data are not publicly available because of privacy and ethical restrictions. The data supporting the findings of this study are available upon request from the corresponding author.
