# Supplementary material for: The Risk Factors for Cervical Cytological Abnormalities Among Women Infected With Non-16/18 High-Risk Human Papillomavirus: Cross-sectional Study
Source: JMIR Public Health Surveill. 2022 Dec 8;8(12):e38628. doi: 10.2196/38628 (PMC9782330; doi:10.2196/38628)
Supplement: Multimedia Appendix 1 [file publichealth_v8i12e38628_app1.pdf]

Appendix Table 1. The main content of the questionnaire.

Age	
Ethnicity	1. Han 2. Other
Education	1. Primary 2. Middle 3. High 4. Graduate
Living address	
Whether there is bleeding during sexual intercourse	1. No 2. Yes
Whether there is abnormal leucorrhea	1. No 2. Yes
menstrual cycle	Duration: _______days<br>Period: _______days
Date of last menstruation	
Whether in menopause	1. No 2. Yes, age of menopause:___<br>3. Uncertainty
Method of contraception	1. No contraceptive methods are used<br>2. Condom<br>3. Contraceptive（___ years)<br>4. intrauterine device (IUD) （___ years)<br>5. Other method
Number of pregnancies	
Number of births	
Family history of cancer	
Have you ever undergone previous cervical cancer screening	1. Yes: Within three years<br>2. Yes: More than three years<br>3. No
History of past tumour	1. No 2. Yes

Appendix Table 2. The original questionnaire (in Chinese).

襄阳市_______区适龄妇女宫颈癌免费检查登记表

姓名：_______　　年龄：_______　　身份证号：_____________________

民族：1. 汉　　2. 其他_______

文化程度：1. 小学及以下　　2. 初中　　3. 高中或中专　　4. 大专及以上

住址：_____省_____县（区）_____乡（街道）_____村（社区）______

**病史情况**		
症状	性交出血	无　　有
	白带异常	无　　有
月经情况	月经周期	持续时间___—___天/周期___—___天
	末次月经	______年______月______日
	绝经	否；是，绝经年龄___岁；不确定
目前使用避孕方法	1. 未避孕　　2. 避孕套　　3. 避孕药___（年）<br>4. 宫内节育器___（年）　　5. 其它避孕方式________	
孕产史	孕___次　　分娩___次	
家族肿瘤史<br>（是否有宫颈癌、乳腺癌、卵巢癌家族史）	无<br>有，如有，请注明：疾病名称____________<br>患者家属与自己的关系：<br>（1）一级亲属（父母、子女、亲兄弟姐妹（同父母））<br>（2）其他，请注明_____________________	
既往接受过宫颈癌筛查　1. 是　①三年内　②三年以上　2. 否		
既往史：其他肿瘤　　1. 无　2. 有，请注明___________		
